# Cost-effectiveness analysis of antimuscarinics in the treatment of patients with overactive bladder in Spain: A decision-tree model

**DOI:** 10.1186/1471-2490-11-9

**Published:** 2011-05-20

**Authors:** Salvador Arlandis-Guzman, Carlos Errando-Smet, Jeffrey Trocio, Daniel Arumi, Javier Rejas

**Affiliations:** 1 Department of Urology, Hospital Universitario La Fe, Valencia, Spain; 2 Female and Functional Urology Department. Fundacion Puigvert, Barcelona, Spain; 3 Pfizer Inc., New York, NY, USA; 4 Pfizer Inc Europe, Alcobendas, Madrid, Spain; 5 Health Economics and Outcomes Research Department, Pfizer España, Alcobendas (Madrid), Spain

## Abstract

**Background:**

Fesoterodine, a new once daily antimuscarinic, has proven to be an effective, safe, and well-tolerated treatment in patients with overactive bladder (OAB). To date, no analysis has evaluated the economic costs and benefits associated with fesoterodine, compared to antimuscarinics in Spain. The purpose of this analysis was to assess the economic value of OAB treatment with fesoterodine relative to extended release tolterodine and solifenacin, from the societal perspective.

**Methods:**

The economic model was based on data from two 12-week, randomized, double-blind, and multicenter trials comparing fesoterodine and tolterodine extended released (ER). Treatment response rates for solifenacin were extracted from the published literature. Discontinuation and efficacy were based on the results of a 12-week multinational randomized clinical trial extrapolated to 52 weeks. Changes in health related quality of life were assessed with the King's Health Questionnaire, which was transformed into preference-based utility values. Medical costs included (expressed in € 2010) were antimuscarinics, physician visits, laboratory tests, incontinence pads and the costs of OAB-related comorbidities, fractures, skin infections, urinary tract infections, depression, and nursing home admissions associated with incontinence. Time lost from work was also considered. Univariate sensitivity analyses were also performed.

**Results:**

At week 12, continents accounted for 50.6%, 40.6% and 47.2% of patients in the fesoterodine, tolterodine, and solifenacin groups, respectively. By week 52, the projected proportions of patients remaining on therapy were 33.1%, 26.5% and 30.8%, respectively. The projected quality- adjusted life years (QALY) gain (compared to baseline) over the 52-week simulation period were 0.01014, 0.00846 and 0.00957, respectively. The overall treatment cost was estimated at €1,937, €2,089 and €1,960 for fesoterodine, tolterodine and solifenacin, respectively. Therefore, treatment with fesoterodine resulted in similar overall costs and greater QALY gain than treatment with either tolterodine or solifenacin. Sensitivity analysis showed that these results were robust to all changes performed.

**Conclusions:**

The results of this economic analysis suggest that fesoterodine is a cost-effective alternative to tolterodine and solifenacin for the treatment of patients with OAB in Spain. Fesoterodine provides additional health benefits while maintain a similar level of costs being a cost-effective treatment strategy from a societal perspective.

## Background

Overactive bladder (OAB) is a symptom-driven condition defined as urinary urgency, with or without urgency urinary incontinence, usually with increased daytime frequency and nocturnal voiding [1,2]. It is a highly prevalent condition, related to an overall OAB prevalence of 11.8% in adults above 18 years of age in Western countries [3]; affecting men (10.8%) and women (12.8%) comparably and increased with age [4]. This corresponds to approximately 1 in 8 adults. These numbers are similar to a 17% prevalence previously reported in 6 European countries (France, Germany, Italy, Spain, Sweden, and the United Kingdom) [4] as well as in the United States in adults above 40 years of age [5]. In Spain, the latest prevalence data showed an OAB and/or urinary incontinence prevalence around 10% in women (between 25 and 64 years), and 5% in men (ages between 50 and 65) [6]. In people over 40, prevalence is set between 20-22%, and higher than 50% over 65 years old [6]. Therefore there should be about 3 million people over 40 suffering this condition in Spain [7].

OAB has devastating consequences for sufferers both genders which impact upon their health related quality of life (HRQoL), self-esteem and relationships [8]. The constellation of OAB symptoms has a profound negative effect on patients' quality of life and general well-being and can affect social, psychological, occupational, domestic, physical and sexual aspects of living [9,10]. Despite the high prevalence of OAB and the significant impact OAB has on patients' daily lives, up to 75% of patients remain untreated [4]. The reasons for this lack of treatment-seeking behaviour include patient embarrassment, the misconception that OAB and urinary incontinence (UI) are natural consequences of ageing, lack of knowledge regarding available treatments and unrealistic expectations [11,12]. OAB often precipitates other medical conditions, such as skin infections, urinary tract infections (UTIs), falls and fractures, and depression [5,13,14]. All these symptoms and facts results in a staggering €4,2 billion in the year 2000 OAB-related healthcare cost in 5 European countries (Germany, Italy, Spain, Sweden, and the United Kingdom); and is predicted to reach € 5,2 billion by 2020, a 25% increase. Incontinence pads were the source of the largest cost, accounting for approximately 63% of the annual per-patient OAB management [15].

A probing article recently stated that successful treatment of OAB depends on persistence with the prescribed medication, and efficacy and tolerability are key influencers of persistence. New antimuscarinic agents are now available for treating OAB that significantly improves symptoms of incontinence, urgency and frequency with few adverse effects. An improved efficacy and tolerability profile should result in greater patient satisfaction and persistence with treatment during long-term therapy [16]. Fesoterodine, a new once daily antimuscarinic, which has been recently marketed in Spain, has proven to be an effective, safe, and well-tolerated treatment in patients with OAB in two large pivotal phase III studies [17,18]. Fesoterodine has also demonstrated clinically and statistically significant improvements health-related quality of life (HRQoL) compared to placebo in subjects with OAB [19].

Health economic analyses assess the implications of projected outcomes and cost of medical interventions. Economic assessments of new therapies are often required by many health decision-making authorities. These are evaluated in order to properly allocate scarce healthcare resources. To date, no analysis has been performed to evaluate the economic costs and benefits associated with fesoterodine related to other existing antimuscarinics in Spain. Thus, the objective of this analysis was to assess the 1-year economic value of OAB treatment with fesoterodine relative to extended release (ER) tolterodine and solifenacin, from the societal perspective.

## Methods

### Economic Model description

**Figure 1:**
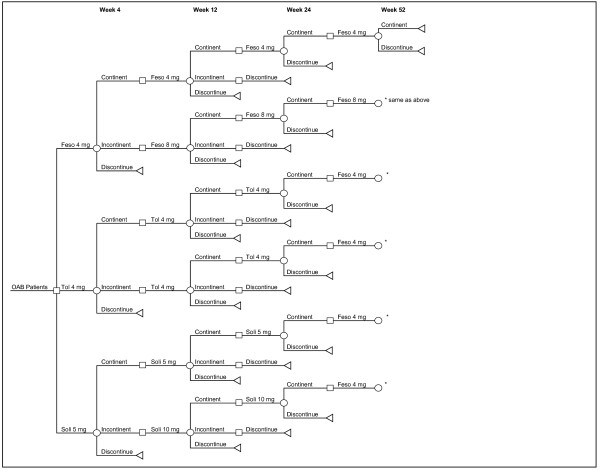
**Decision tree model**.

A cost-effectiveness analysis (CEA) was performed in this study through a decision-tree model developed in an Excel spreadsheet. Cost-effectiveness is typically expressed as an incremental cost-effectiveness ratio (ICER): the ratio between the difference in costs and the difference in benefits of two interventions. A threshold value is often set by policy makers, who may decide that only interventions with an ICER below a specific threshold are cost effective, although decision on funding may be more complex and subject to additional factors. In Spain, there is no a generally accepted cost-effectiveness threshold value. However, ICER below €30,000 per Quality-adjusted-life-year (QALY) gained use to be considered cost-effective [20]. The decision-tree model was developed to simulate the typical clinical treatment pathway of an individual initiating OAB therapy with fesoterodine 4 mg/day, ER tolterodine 4 mg/day, or solifenacin 5 mg/day (see Figure 1). This economic model assessed the economic benefits of treating OAB with incontinence with fesoterodine relative to extended release tolterodine or solifenacin, based on data from two 12-week, randomized clinical trials and the published literature (see ahead). At weeks 4, 12, 24 and 52 patients were classified as responders (those who are restored continence or <1 urge urinary incontinence episode/24 hrs) or non-responders. At four weeks after treatment initiation, treatment responders are assumed to continue their initial therapy. Non-responders are assumed to titrate to the higher dose of fesoterodine or solifenacin. As an assumption, 50% of responders to fesoterodine 4 mg and solifenacin 5 mg were assume to titrate to the higher dose at week 4, and the same proportion of non-responders in both treatments were elect not to titrate. Because only one dose of tolterodine is modelled, all non-responding patients are assumed to continue with the same treatment. This allows for patients in the tolterodine arm to have two chances at treatment response, as there are for fesoterodine and solifenacin. Responder status is assessed again at week 12. At this point, responders are assumed to continue treatment and all non-responders are assumed to discontinue (i.e., no further titration is allowed and patients are not assumed to lower previously-titrated doses). Responders to treatment at week 12 are assumed to remain responders for the duration of the model unless they discontinue for non-efficacy reasons. This assumption is supported by studies of tolterodine that suggest very little change in treatment efficacy takes place over the longer term in patients who are compliant with therapy [21,22]. Therefore, no further changes in drug efficacy were assumed to occur after week 12 although some patients were assumed to discontinue for non-efficacy reasons between weeks 12 and 52 [23,24]. Discontinuation, efficacy, and changes in HRQoL results of the trials were extrapolated from 12 to 52 weeks using the method of last observation carried forward for all the comparators.

Following Spanish CEA guidelines, the comparators considered in the analysis should be the relevant ones in current clinical practice; i.e. the most commonly used in the higher number of patients [25]. The 96% of the I.M.S. reported Spanish antimuscarinic sales [26] are attributed to fesoterodine, tolterodine and solifenacin; the three most consumed drugs in Spain; with the rest of antimuscarinics commercialized in Spain included in the remaining 4% of the OAB treatment market, and are not considered in this study due to its low economic impact on health budgets. As the time horizon for this analysis is 1 year, no discount rate was included in the analysis [25]. As this analysis was not carried out on humans, it represents a hypothetical patient ant the treatment pathway simulation, it was not needed to get the approval of any ethics committee.

### Clinical trial data

Model is populated with data from more than 1.000 patients with OAB and incontinence enrolled in two 12-week, randomized, placebo controlled, double blind, international clinical trials [17,18]. These phase III trials were designed to investigate the efficacy, tolerability, and safety of fesoterodine 4 mg/day, fesoterodine 8 mg/day, tolterodine 4 mg/day extended released, and placebo in patients with OAB and incontinence. Male and female subjects ≥18 years of age with ≥8 micturitions/24 h, OAB symptoms with urinary urgency and ≥1 urge urinary incontinence episode/24 h were included in these analyses. Trial evaluation endpoints were at weeks 2, 8, and 12 post-treatment starts-up. To estimate the treatment response rates at week 4, the efficacy and discontinuation data at week 8 were chosen as a proxy. As solifenacin was not a comparator in either clinical trial, efficacy data (5 mg and 10 mg data combined) were abstracted from clinical trial published in the literature [27-30] and enrolling patients with a similar profile than the ones enrolled in tolterodine and fesoterodine trials. HRQoL data was extrapolated from the interventions in the clinical trial. Four studies published the endpoint of solifenacin restoration of continence. Haab et al. [27] reported that 52% of patients on either solifenacin 5 mg or 10 mg were restored to continence at week 12. However, this group did not report the corresponding value for the placebo group. Millard and Halaska [28] reported restoration of continence for those with severe incontinence at baseline (15.3%, 28.4%, and 30.5% for placebo, solifenacin 5 mg, and 10 mg, respectively). Wagg et al. [31] reported 49.1% and 47.3% of elderly patients on solifenacin 5 mg and 10 mg, respectively, and 28.9% of patients on placebo had restoration of continence. Because fesoterodine trial populations include non-elderly subjects, the analysis of phase III clinical trials by Cardozo and colleagues was chosen to make assumptions on such segment of population [29]. This study examined data from 2,030 incontinent subjects. Of patients incontinent at baseline, 34% were restored to continence at week 12, and 51% and 52% were restored to continence in the solifenacin 5 mg and 10 mg groups, respectively. These data correspond to a relative risk (RR) of 1.50 and 1.53, respectively, compared to placebo. We applied these RRs to the placebo values at weeks 12 to obtain an estimate of the solifenacin efficacy. Efficacy at week 2 or week 8 is also assumed to be the RR multiplied by the corresponding placebo value. Efficacy values, measured as percentage of patients with resolution of incontinence, are shown in Table 1.

**Table 1:** Efficacy (% resolution of incontinence) and discontinuation data included into the economic model

% Resolution of incontinence (<1 episode/24 hrs)	W2	W8	W12	W12|W8	W12|~W8	
Placebo	20,99%	34,66%	27,84%	80,33%	14,16%	
Fesoterodine 4 mg	30,41%	50,00%	43,67%	87,34%	21,05%	
Fesoterodine 8 mg	40,59%^a^	55,38%^a^	50,21%	90,65%	24,20%	
Tolterodine	29,23%	49,47%	38,18%	77,17%	13,04%	
Solifenacin 5 mg^c^	31,49%	51,99%	41,76%	80,33%^d^	21,24%	
Solifenacin 10 mg^e^	32,11%^a^	53,01%^a^	42,58%	80,33%^d^	21,66%^b^	

**Other data**	**% Nocturia *(> = 2 episodes/night) *% of patients with nocturia at baseline without nocturia at W12**			**Constipation**		

Placebo	54,34%			1,99%		
Fesoterodine 4 mg	55,56%			4,15%		
Fesoterodine 8 mg	55,90%			6,01%		
Tolterodine	57,69%			2,76%		
Solifenacin 5 mg	54,34%^i^			3,53%^j^		
Solifenacin 10 mg	88,72%^j^			7,72%^j^		

**Discontinuation**	**W2**		**W8**	**W12**	**W24**^**f**^	**W52**^**f**^

Placebo	100%		92,84%	90,64%	79,32%	59,27%
Fesoterodine 4 mg	100%		92,67%^g^	equal to placebo data ^h^		
Fesoterodine 8 mg	--		--	equal to placebo data ^h^		
Tolterodine	100%		95,76%	equal to placebo data ^h^		
Solifenacin 5 mg	100%		94,06%	equal to placebo data ^h^		
Solifenacin 10 mg	--		--	equal to placebo data ^h^		

The symbol "|" denotes conditional response rates; the response at week 12 given response at week 8. The symbol "~" denotes non-response; no-response at week 12 given no-response at week 8

Source: Fesoterodine phase III trials, except when a different source is described [17,18].

a - values not used in the decision tree; only used to calculate conditional response rates

b - conditional response rates estimated by applying the W8 low dose: high dose response ratio to the W12|~W2(~W8) non-titrating response rate

c - equal to 1,5 times the placebo rates [29]

d - conditional response equal to placebo rate

e - equal to 1,5294 times the placebo rates [29]

f - W24 and W52 values were extrapolated from clinical trial data using an exponential decay curve fitted to W2, W8, and W12 discontinuation values

g - discontinuation rate of fesoterodine 4 mg at W8 in the trial data was greater than that of placebo at W12, therefore this value was set equal to the W12 rate.

h - discontinuation rates at week 12 were found to be not statistically significant for any treatment [30], therefore the values are equal to placebo discontinuation

i - relative rates from published literature [42]; solifenacin 5 mg not found to be significantly different than placebo; solifenacin 10 mg 63.28% higher than placebo

j - relative rates from published literature [29]; solifenacin 5 mg 1,778 times placebo; solifenacin 10 mg 3,889 times higher than placebo

Some published data have not shown 12-week discontinuation rates for antimuscarinics to be significantly different than placebo [30]. Therefore, the values considered in this analysis are equal to placebo discontinuation (Table 1). Fesoterodine phase III clinical trials were also the source for the percentage of patients with nocturia at baseline (48.58%) and for the percentage of patients with nocturia at baseline without nocturia at W12 is shown in Table 1.

### Costs

The cost, measured in Euros, of each treatment arm is the sum of purchased medical and non-medical resources used and non purchased resources (lost productivity of the patient or unpaid family member/caregiver support). Both direct and indirect costs related to OAB were considered in the basecase analysis following a societal perspective that is recommended in Spanish CEA guidelines [25]. Additionally, the payer perspective (Spanish National Healthcare system, which considered only direct costs) was obtained and presented separately as a sensitivity analysis [25]. Costs inputs were taken from the published literature and expressed in €2010. Direct medical costs included were antimuscarinic drugs, physician visits, laboratory tests, incontinence pads, and costs of OAB- or incontinence-related co morbidities (fractures, skin infections, urinary tract infections, depression, and nursing home admissions all of them associated with incontinence), and the cost of treating constipation adverse events (Table 2). Health care resource utilization included is showed in Table 3.

**Table 2:** Direct medical and productivity costs included into the fesoterodine economic model

Resource costs	Costs	Source
Cost per incontinence pad	0.58 €	[43]
Cost of general practitioner visit	26.78 €	[44]
Cost of specialist visit	58.60 €	[44]
Cost of laboratory tests (urinalysis)	2.56 €	[44]
Constipation cost/day^a^	0.16 €	[44]
Fesoterodine 4 mg (cost/day, with taxes)	1.70 €	[43]
Fesoterodine 8 mg (cost/day, with taxes)	2.72 €	[43]
Tolterodine ER (cost/day, with taxes)	1.70 €	[43]
Solifenacin 5 mg (cost/day, with taxes)	1.67 €	[43]
Solifenacin 10 mg (cost/day, with taxes)	2.67 €	[43]
Fracture	5,742.8 €	[45]
Skin Infection episode	53.1 €	[44]
Urinary Tract Infection episode	53.1 €	[44]
Depression (€/patient/year)	2,699 €	[46]
Nursing Home	14,831.4	[44]
Average hourly wage	13.51 €	[47]
Average number of hours worked per week	40	[47]
% Employed in population	59.83%	[47]
Decrease in hours worked due to incontinence ^b^	21.1%	[33]
Reduced daytime productivity due to nocturia ^c^	9.2%	[34]

^a^The mean cost/day per patient of managing constipation includes a daily dose of oral laxative. ^b^Women without incontinence report working 38 hours/week vs. 30 hours for women with incontinence. ^c ^13.8% work impairment for patients with nocturia vs. 4.61% impairment for controls.

**Table 3:** Healthcare resource utilization and other data included into the fesoterodine economic model

Input		Controlled (continent)	Uncontrolled (incontinent)	Untreated (incontinent)	Source
Proportion using incontinence pads		0%	67%	67%	[24]
Number of incontinence pads/day		0	4.23	4.23	[48]
Number of general practitioner visits/month		0.133*	0.2	0.2	[48]
Number of specialist visits/month		0.117*	0.15*	0.15	[48]
Number of laboratory tests/month		0.033*	0.078*	0.078	[48]
OAB-related Co morbidities: rate per year					

Fracture: 6-month probability of a fall with fractures (4% decrease in utility values [49]**)		2.5%	5.3%	5.3%	[48,50]
Skin infection: 6-month probability		10.7% (0.3 infections per person in entire population) (2.8 events/affected patient)	9.3% (0.6 infections per person in entire population) (6.5 events/affected patient)	9.3%	[48]
UTI: 6-month probability		19% (0.3 infections per person in entire population) (1.6 events/affected patient)	30.7% (0.7 infections per person in entire population) (2.3 events/affected patient)	30.7%	[48]

Depression (48% decrease in utility values [51])***	% female in clinical trial data	-	80.87% OAB w/UUI****	-	[52]
	Women	9.10%	18.90%	-	[52]
	Men	4.30%	18.60%	-	[52]
	Overall	8.08%	18.84%	-	[52]
	
Nursing home: Admission rate per 1000 patient-years	Women	31	73	73	[53]
	Men	24	98	98	[53]
	
(% decrease in utility values [49])*****	Overall	29.5	78.3	78.3	[53]

Utility values [17,18]		0.9569	0.9412	0.9332 (baseline value)	

*Bolge et al. 2006, showed that successfully-treated patients saw a GP 25% fewer times than unsuccessfully-treated patients. Additionally, the mean number of non-GP visits in past 6 months was 0.9 and 0.7, respectively for incontinent and continent patients. Bolge et al 2006 also showed the following number of urinary test: 0.47 and 0.2 urine tests/patient over last 6 months, respectively for incontinent and continent patients. **Reported wrist fracture utility was 0.96. Assumed 0.7 utility for 7 weeks, referenced from National Osteoporosis Foundation review. ***Women with urinary incontinence with major depression have utility of 0.45. Women with urinary incontinence without major depression have utility of 0.86. 0.45 represents a 48% reduction from 0.86. This means a patient experiencing depression would have 48% reduction in QALY gain than a comparable patient experiencing no co morbidities. ****By using the proportion of women and men in the trial data (80.87% women, 19.13% men), we calculated the weighted average of the above data for inclusion into the model. *****There are no published data to provide the difference in utility of OAB patients in and out of nursing homes. One estimate is to use the utility value of nursing home admission due to hip fracture provided in the UK HTA (0.4), but this value would be an overestimation of the utility decrement for our OAB population since patients who enter the nursing home due to a hip fracture are in much worse physical condition (and, it follows, utility) than patients entering a nursing home due to OAB and/or incontinence. For this reasons, no decrease in utility value is considered.

The probability of OAB-related co morbidities depended on the patients' responder and treatment status. Studies have shown the risk of falls and fractures, skin infections, urinary tract infections, to be positively associated with OAB and the risk of depression and nursing home admissions to be associated with OAB with incontinence. Patients with controlled OAB have lower risk of co morbidities than non-responders or untreated patients (Table 3). Skin infections and UTIs have the possibility of occurring more than once per year, and the expected number of events per person is included. The mean numbers of UTI per patient for these two populations are 0.3 and 0.7, respectively. To calculate the average number of UTIs per patient experiencing one, the following formula was used (a representative calculation for successfully-treated patients is shown):

Analogously, we calculate the expected number of UTIs per unsuccessfully-treated patient as well as the expected number of skin infections for both treatment categories. The utility decrements for fracture, depression, and nursing home are also considered in the analysis (Table 3), with its corresponding literature sources.

An assessment of the indirect productivity costs associated with OAB and incontinence was also included. Lost productivity at work due to OAB can come from many sources. For one, frequent voiding during sleep time (nocturia) can deprive one of needed sleep, mimicking the symptoms of insomnia. We assume the decreased productivity during work hours for those with nocturia relative to those without nocturia to be 9.2% [32], representing the difference between the percent work impairment for patients with nocturia (13.8%) and control patients (4.61%), Table 2. Another cause for lost work is the presence of incontinence, where patients may choose to work fewer hours because of their condition. Data show women who work for pay report working fewer hours per week (38 vs. 30) [33]. Incontinent men with OAB factor their symptoms into decisions about location and hours worked more than continent men or women with OAB and more than twice as much as women with incontinence (21% vs. 8%). A conservative assumption to capture the monetary value of this productivity loss would be to assume those incontinent patients who are employed work 21% fewer hours than those without incontinence (the percent difference between the number of hours worked per week for women without incontinence versus women with incontinence [38 hours-30 hours]/38 hours). Spanish employment and wage data were entered into the model to estimate the decreased productivity while at work due to interrupted sleep by nocturia episodes and lost time from work due to incontinence (Table 2). The model inputs the productivity costs of each treatment arm compared to no-treatment, as relative productivity gains. As explained, incontinence and nocturia episodes were related, respectively, with reduced work hours and lost productivity while at work and their associated costs. If no treatment is considered, higher productivity costs are observed compared to receiving an adequate treatment. For this reason, negative values (or productivity gains) would be obtained for each treatment arm compared in this study.

### Effectiveness

In this analysis, effectiveness of medical interventions was expressed in term of Quality-Adjusted-Life-Years (QALY) gain. Changes in HRQL were assessed from a disease specific HRQL tool during the trial: the King's Health Questionnaire [34], which was transformed into preference-based utility values for responders and non-responders [35]. KHQ is an instrument specifically designed to assess the impact of bladder problems on HRQL in women and has been shown to be reliable, internally consistent and valid in men and women [34]. The items of the KHQ cover 5 domains: role limitations; physical functioning; social functioning; emotional problems; sleep disturbance and general health. Each patient's 5 domain scores from the KHQ were incorporated into a regression algorithm developed by Brazier et al. to generate preference-based utilities [35]. The authors have previously used this approach to estimate preference-based utilities from the SF-36 [36]. KHQ responses were collected from trial participants at baseline and again at week 12 (Table 3). Utility for patients not on therapy was assumed equal to the baseline value. The QALY gains from baseline to week 12, from week 12 to week 24, and from week 24 to week 52 were calculated based on patient's treatment status (continent, incontinent, or no treatment). The difference between week 52 utilities and baseline utilities for responders and non-responders were the effect measure in the cost-effectiveness ratio. As described in Table 3, fracture, depression, and nursing home admissions are assumed to be associated with a decrement in utility relative to not having these conditions. The percent decrease is included as a model condition.

### Cost-effectiveness analysis (CEA)

For each of the treatment interventions, the model calculates the expected total one-year costs (including both direct and indirect costs). The model also determines the expected proportion of patients on each treatment having restoration of continence at the end of 1 year and the QALYs gained for each intervention. These outputs are combined to create the incremental cost effectiveness ratio (ICER), representing the additional cost associated with fesoterodine treatment divided by the additional QALYs gained with fesoterodine treatment relative to tolterodine and solifenacin, respectively.

### Sensitivity analysis

Several univariate sensitivity analyses of fesoterodine versus tolterodine ER or solifenacin were performed in order to find if the results were robust to changes in main assumptions or inputs of the analysis: trial time horizon, co morbidities costs and rate per year, utility estimates, direct medical services costs, utilization and productivity data, proportion of continent patients at 12 and 52 weeks, and proportion of responders to fesoterodine 4 mg and solifenacin 5 mg who elect to titrate to the higher dose at week 4, as well as the proportion of non-responders who elect not to titrate. All the variables were changed in a range of ±25%, a plausible range of variation of both costs and health effects. Following Spanish CEA guidelines [25], the payer perspective results (Spanish National Healthcare system, which considered only direct costs) were obtained and presented separately as a sensitivity analysis.

## Results

**Figure 2:**
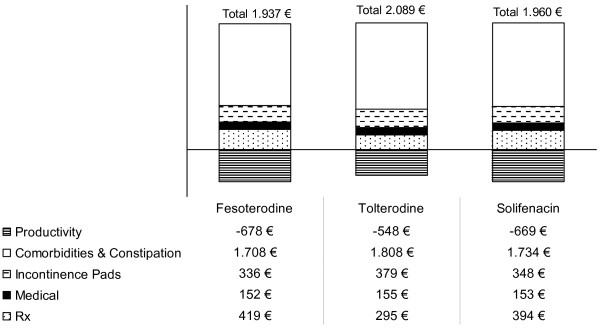
**Cost outcomes of the OAB economic model by drug (base case scenario)**.

Clinical and cost-effectiveness results are shown in Table 4 and Figure 2. At week 12, the proportion of continent patients on each treatment at the end of the model period (responders) was higher for fesoterodine and solifenacin than for tolterodine. By week 52, the projected proportions of patients remaining on therapy was again higher in the fesoterodine arm of the study. The projected quality- adjusted life years (QALY) gain (compared to baseline) over the 52-week simulation period showed greater gain for fesoterodine in comparison with the other two drugs evaluated, while the overall treatment costs were similar among the three drugs (Figure 2). However, as the analysis was deterministic, did not allow for a statistical comparison between drugs. These results are related to the lower total treatment costs of fesoterodine in 52 weeks due to the proportion of responder's patients continuing therapy throughout the 52-week period. Therefore, treatment with fesoterodine resulted in lower overall costs and greater QALY gain than treatment with either tolterodine or solifenacin.

**Table 4:** Outcomes of the OAB economic model for the base-case scenario

Treatment	Continent at Week 12	Continent at Week 52	QALY gain	**Total costs (**€**)**	ICER
**Fesoterodine**	50.6%	33.08%	0.01014	1,937	-

**Tolterodine**	40.6%	26.53%	0.00846	2,089	Cost-saving

**Solifenacin**	47.2%	30.85%	0.00957	1,960	Cost-saving

ICER = Incremental cost-effectiveness ratio

Sensitivity analysis showed that, for the majority of plausible scenarios, the results obtained were robust to all changes performed in the inputs data considered, consistently showing similar cost but higher effectiveness for fesoterodine over the other compared antimuscarinics (cost-saving ICERs), except when the time horizon was changed to 12 weeks. Nevertheless, if the total duration of treatment were reduced to approximately 3 months only, fesoterodine would still continue being a cost-effective option, with an incremental cost-effectiveness ratio of 574€ and 14,568€ compared to tolterodine and solifenacin, respectively when the NHS perspective is analyzed only (Table 5). If the societal perspective is considered, then, fesoterodine would not be cost-effective relative to solifenacin for treatment duration of 12 weeks or less only. On the other hand, changing proportions of continent subjects modified the ICERs of fesoterodine relative to the other comparators only when the percentages were reduced by a 25% (table 5). However, ICERs of fesoterodine over tolterodine and solifenacin were, respectively, of €39,447 and €17,814 per QALY gained, which are still in the range of acceptable cost-effectiveness in Spain.

**Table 5:** Univariate sensitivity analyses of fesoterodine versus tolterodine ER or solifenacin

Assumption changed	**Fesoterodine ICER relative to**:	
	
	Tolterodine	Solifenacin
**Base-case scenario**: Results weeks 52, societal perspective	Cost-savings	Cost-savings
Weeks 52, health system perspective	Cost-savings	Cost-savings
Weeks 12, health system perspective	€574	€14,568
Weeks 12, societal perspective	€9,106	€216,316
+/- 25% in OAB-related co morbidities costs	Cost-savings	Cost-savings
+/- 25% in OAB-related co morbidities rate per year in continent patients	Cost-savings	Cost-savings
+/- 25% in OAB-related co morbidities rate per year in incontinent patients	Cost-savings	Cost-savings
+/- 25% in utility estimates	Cost-savings	Cost-savings
+/- 25% in % of responders to fesoterodine 4 mg and solifenacin 5 mg that titrate to the higher dose at week 4	Cost-savings	Cost-savings
+/- 25% in % of non-responders to fesoterodine 4 mg and solifenacin 5 mg that do not to titrate to the higher dose at week 4	Cost-savings	Cost-savings
+25% in % continent patients at week 12	Cost-savings	Cost-savings
- 25% in % continent patients at week 12	€39,447	€17,814
+/- 25% in % continent patients at week 52	Cost-savings	Cost-savings
+/- 25% in medical services costs*	Cost-savings	Cost-savings
Medical services utilization	Cost-savings	Cost-savings
+/- 25% % of incontinent patients using pads	Cost-savings	Cost-savings
+/- 25% No. pads/day for incontinent patients	Cost-savings	Cost-savings
+/- 25% in # GP visits/month	Cost-savings	Cost-savings
+/- 25% in # specialist visits/month	Cost-savings	Cost-savings
+/- 25% in # lab tests/month	Cost-savings	Cost-savings
Productivity data	Cost-savings	Cost-savings
+/- 25% in decrease in hours worked due to incontinence	Cost-savings	Cost-savings
+/- 25% in reduced daytime productivity due to nocturia	Cost-savings	Cost-savings
+/- 25% in % employed in population	Cost-savings	Cost-savings
+/- 25% in average hourly wage	Cost-savings	Cost-savings

ICER = Incremental Cost-effectiveness Ratio. *Costs of incontinence pads, general practitioners visits, specialist visits, laboratory tests and constipation cost/day.

## Discussion

This decision-tree model cost-effectiveness analysis, the first one that compares fesoterodine and other salient antimuscarinics in Spain, showed that the treatment with fesoterodine resulted in similar overall costs but greater QALY gain than treatment with either tolterodine or solifenacin. In other words, fesoterodine acquisition cost is outweighed by the lower costs related to both direct and indirect resources used considered in this study. This analysis tried to be as much robust as possible, including the relevant comparators (the ones that represent 96% of the Spanish year 2010 OAB treatment market), the adequate efficacy data and the adequate costs. Additionally, several univariate sensitivity analyses were done, to demonstrate the strength of the assumptions. As explained in the results section, fesoterodine continued being the dominant option in most sensitivity analyses done, particularly when the time horizon of analysis is set at 52 weeks. When the time horizon is changed to 12 weeks only (this happening in some particular patients who abandon the OAB treatment rapidly), fesoterodine would still be a cost-effective option when the NHS perspective is considered only: incremental cost-effectiveness ratios per QALY gained of 574€ and 14,568€ compared to tolterodine and solifenacin, respectively. However, if it is considered the societal perspective in the 12-weeks scenario, fesoterodine would be cost-effective relative to tolterodine only. Nevertheless, the plausibility of this scenario is really low even considering than persistence rate with antimuscarinics has been showed to be lower than with other treatments for chronic conditions [16]. At least, in the case of solifenacin persistence, it has been communicated to be above 90% after 12 weeks of starting the therapy [27].

The model is considered to be comprehensive by including several aspects of OAB affecting the overall economic burden of disease. These include the cost of incontinence pads and the cost of lost productivity due to impairment at work or lost time at work due to OAB symptoms. Also, it includes the cost of associated co morbidities arising with uncontrolled OAB. The main reason of incorporating indirect costs is due to their relevance for OAB. Kobelt et al (2003) [32] conclude that in an otherwise healthy and professionally active group of individuals, waking at night to void, significantly diminishes their overall well-being, vitality and productivity, leading to a significant level of indirect and intangible costs. Additionally, over 21% of the OAB population worried about interrupting meetings with frequent trips to the toilet and 3% of the population changed jobs or were fired because of their bladder control problems [37]. Considering these figures, lost productivity and lost wages were included in this analysis. Nevertheless, results are consistent with base case scenario even if indirect costs are not included in the analysis and the perspective of National health System is analyzed solely.

As in all scientific works, this analysis includes limitations and strengths. One of the strengths of the model is the use of the clinical trial data for the comparison of all alternatives, which ensures comparable patient populations among the treatments. However, unlike in the actual trial, the model assumes patients whose OAB is not controlled by treatment at each assessment point will discontinue therapy at that point, replicating a treatment pattern more reflective of real clinical practice. On the other hand, solifenacin was not included in the clinical trial and was modelled using published data. In order to diminish the possible impact in the results of solifenacin data, the meta-analysis of phase III clinical trials by Cardozo and colleagues was chosen [29], which examined data from 2,030 incontinent subjects. Inclusion criteria for these studies were rather similar to the fesoterodine trials. Another possible limitation is that our analysis was not able to incorporate the so-called out-of-pocket expenses, i.e.: incontinence pads not funded by the NHS, particularly because studies used as a sourcing data for the analysis were unable to differentiate it. Finally, this economic model was designed as deterministic, then; only point estimates are showed not allowing for statistical comparison between drugs in both costs and effectiveness. However, these results encourages for development of more sophisticated economic modelling of such drugs using, i.e., probabilistic approach.

As previously mentioned, this is the first cost-effectiveness analysis performed with Spanish data that compares fesoterodine with another antimuscarinics highly used in our health context. However, several economic analyses have been previously published with different antimuscarinic drugs in other settings. Considering the comparators included in this analysis, four economic evaluations have compared solifenacin to ER tolterodine in three different settings [38-41]. These analyses showed that treatment with solifenacin was less costly and more effective than tolterodine. Only one of the four economic evaluations includes fesoterodine as a comparator [41], with controversial findings in comparison with the results include in our work. The article included some information based in the experience in clinical practice use of all antimuscarinics except for fesoterodine since it was not marketed yet at the time of the analysis included in the paper (year 2008). Due to the lack of available data, fesoterodine inputs were based on extrapolated assumptions from the use of other antimuscarinics instead of data from clinical trials. Another difference is the type of resources and corresponding cost considered in the analysis, since only were considered some direct medical costs, while in the present economic analysis, following Spanish guidelines, indirect costs were also included [25]. Drug-specific treatment persistence data were obtained from the Information Management System database and covered the 12-month period ending in April 2008. As fesoterodine was only on the market since July 2008, treatment persistence data from longitudinal databases were not yet available. To approximate the percentage of patients stopping and switching treatment with fesoterodine in the base-case analysis, Cardozo et al. [41] used the persistence rates for tolterodine ER, which could potentially represent a bias against fesoterodine. Finally, the percentage of patients who stop or switch treatment due to poor compliance was based on expert opinion.

## Conclusions

In conclusion, the results of this economic analysis, supported by sensitivity analyses and despite the limitations mentioned, suggest that fesoterodine is a cost-effective alternative to tolterodine and solifenacin for the treatment of patients with OAB in Spain. Fesoterodine provides additional health benefits while maintain a similar level of costs being a cost-effective treatment strategy from a societal perspective and from the National Health System as well.

## Competing interests

This study was funded by Pfizer, Inc. DA and JR are employees of Pfizer Inc Spain and JT is employee of Pfizer Inc, New York. SA and CES have not received any financial support from Pfizer Inc for writing or interpreting the present research. SA and CES declare that they do not have financial and non-financial competing interests. A funding was received by MEISYS, an independent consultancy agency, for drafting the manuscript and also for final adaptation of the model to the Spanish context.

## Authors' contributions

SA, CES and JR participated in the design of the study, coordination, carried out the acquisition of data and revised it critically for important intellectual content. JT developed the decision-tree model for local adaptations, which was used to obtain the results of this study. All authors have made a significant contribution to the findings and methods in the paper, and interpretation of data. All authors helped to draft the manuscript, read, modified and approved the final manuscript.

## Pre-publication history

The pre-publication history for this paper can be accessed here:

http://www.biomedcentral.com/1471-2490/11/9/prepub
